# Ability of adhesion and biofilm formation of pathogens of periprosthetic joint infections on titanium-niobium nitride (TiNbN) ceramic coatings

**DOI:** 10.1186/s13018-020-01613-w

**Published:** 2020-03-04

**Authors:** Alessandro Bidossi, Marta Bottagisio, Roberta De Grandi, Elena De Vecchi

**Affiliations:** IRCCS Orthopedic Institute Galeazzi, Laboratory of Clinical Chemistry and Microbiology, Via R. Galeazzi, 20161 Milan, Italy

**Keywords:** Prostheses and implants, Hypersensitivity, Prosthesis-related infections, Biofilms, Metal ceramic alloys

## Abstract

**Background:**

Orthopedic metal implants are notoriously associated with release of metallic ions able to cause biological adverse reactions which might lead to implant loosening and failure. To limit any possible adverse reactions, ceramic coatings for orthopedic metal implants have been introduced. However, information regarding the interaction of these coatings with microbes responsible for periprosthetic joint infections (PJIs) is lacking. Hence, the aim of the present in vitro study is to assess the microbial affinity to a titanium-niobium nitride (TiNbN) coating.

**Methods:**

Adhesion and biofilm formation of clinical isolates of *Staphylococcus aureus*, *Staphylococcus epidermidis*, *Pseudomonas aeruginosa*, and *Cutibacterium acnes* were assessed on TiNbN-coated titanium discs in comparison with uncoated titanium and cobalt-chrome alloys discs, with either smooth or rough surfaces. Bacterial adhesion was performed by counting adhered bacteria in the first hours of incubation, and the biofilm formation was performed by means of a spectrophotometric assay and by confocal laser scan microscopy after 72 hours of incubation.

**Results:**

Overall, *Staphylococcus aureus* and *Staphylococcus epidermidis*, among the most common bacteria responsible for PJIs, displayed a significantly decreased attachment in the first hours of contact and, when cultured in presence of TiNbN coating, in comparison with CoCrMo. Biofilm formation of the four tested strains was comparable on all alloys.

**Conclusions:**

Although the onset of a PJI is more complex than in an in vitro scenario, these findings suggest that TiNbN-coated orthopedic implants do not increase PJIs risk while ameliorating tribological and surface properties could represent a valid choice to limit possible complications such as metal hypersensitivity.

## Background

Orthopedic metal implants are associated with the release of metal ions, which, in some cases, might lead to complications after joint arthroplasties. Heavy metals such as nickel (Ni), chromium (Cr), and cobalt (Co) are known sensitizers able to trigger the host immune system. Indeed, through the formation of protein complexes, they might cause hypersensitivity reactions, which could possibly lead to implant loosening and bone resorption with consequent failure and revision of the implant [1, 2]. Thus, to limit any possible adverse local or systemic reactions, hypoallergenic coatings have been introduced in orthopedic implants [3]. In this scenario, ceramic coatings like titanium nitride (TiN) used for joint implants, especially for knee prostheses, exhibited favorable biocompatibility, physical, and tribological properties comparable to uncoated conventional prosthesis [4, 5]. Therefore, the bacterial affinity to newly developed biomaterials should be carefully evaluated. Indeed, prosthetic joint infections (PJIs) are among the most common and feared complications in orthopedic surgery [6]. Bacterial colonization principally relies on the ability to adhere to abiotic surfaces and to produce of an exopolymeric matrix called biofilm. Staphylococci dominate PJIs etiology with coagulase-negative staphylococci (CoNS) overcoming *S. aureus* in European countries, followed by Gram-negatives and anaerobes like *Cutibacterium acnes* [7, 8].

With the aim to improve the properties of TiN coating, a novel titanium-niobium nitride (TiNbN) has been proposed as an enhanced ceramic coating for biomedical applications [9]. Hence, the aim of the present in vitro study was to evaluate the microbial affinity to this ceramic coating by evaluating bacterial adhesion and biofilm formation in comparison with uncoated titanium and cobalt-chrome alloys, commonly used to manufacture prosthetic devices.

## Methods

### Experimental conditions

The in vitro activity of a commercially available TiNbN coating (Bioloy®, Permedica S.p.A., Merate, Italy) was tested on clinically relevant strains isolated at the Laboratory of Clinical Chemistry and Microbiology of the IRCCS Galeazzi Orthopedic Institute (Milan, Italy). In particular, biofilm-producer clinical isolates of *Staphylococcus aureus*, *Staphylococcus epidermidis*, *Pseudomonas aeruginosa*, and *Cutibacterium acnes* were used in this study. The adhesion and biofilm production of the aforementioned bacterial species were tested on the following substrates: Ti6Al4V alloy (ISO 5832/3), CoCrMo alloy (ISO 5832/12), and Ti6Al4V alloy (ISO 5832/3) coated with TiNbN (Bioloy® coating). Discs of 22-mm diameter and 5-mm thickness were manufactured with these three different substrates and finished with two different surface roughness features. A sandblasted finished surface with a mean roughness of Ra = 4 μm (rough surface) to resemble the sandblasted surface of prosthesis component adjacent to bone (e.g., external part of acetabular cup, femoral stems) and a mirror polished surface with a mean roughness of Ra = 0.03 μm (smooth surface) to resemble the mirror-finished surfaces of the sliding components of prosthesis (e.g., tibial tray, femoral head). All discs were provided by Permedica S.p.A. (Merate, Italy).

### Evaluation of bacterial adhesion

In order to evaluate if the physical properties of TiNbN coatings (e.g., electrostatic interactions, hydrophilicity, hydrophobicity) might have an impact on the adhesion of the bacterial cells during the first contacts with the surface, titanium alloy (Ti6Al4V), CoCrMo, and Bioloy®-coated discs were incubated with bacteria for 30, 60, and 120 min, as previously described [10]. Specifically, discs were placed in 6-well microplates containing 6 ml of either brain heart infusion (BHI, Biomérieux, Marci l’Etoile, France) broth or thioglycollate (THI, Biomérieux, Marci l’Etoile, France) for anaerobes. A bacterial overnight culture was suspended at a density of 0.5 McF (approximately 1.5 × 10^8^ CFU/ml) and diluted to a final concentration of approximately 1.0 × 10^6^ CFU/ml per wells. After 30, 60, and 120 min of incubation, discs were rinsed three times with sterile saline to remove non-adherent bacteria. Then, discs were immersed in 5 ml of 0.1% w/v dithiothreitol (DTT; Sigma-Aldrich, Milan, Italy) and mechanically stirred for 15 min at room temperature to detach bacteria adhered to the discs. Dilutions of the obtained fluids were plated onto tryptic soy agar (TSA) or Schaedler agar (SCH) and incubated at 37 °C in aerobic (staphylococci and *P. aeruginosa*) or anaerobic (C*. acnes*) atmosphere for 24 h for CFU count. Each strain was tested in triplicate.

### Semi-quantitative analysis of biofilm by spectrophotometric assay

The amount of biofilm formed on the surface of each disc was determined by the spectrophotometric assay proposed by Christensen et al. [11]. Briefly, according to the previously described experimental conditions, Ti6Al4V, CoCrMo, and Bioloy®-covered discs were placed in 6-well microplates in triplicate with a bacterial suspension of 1.0 × 10^6^ CFU/ml. To meet specific bacterial demands, *C. acnes* was cultured in an anaerobic atmosphere in THI broth, while staphylococci and *P. aeruginosa* in BHI broth in aerobic conditions. After 72 h of incubation, the growth medium was removed; discs rinsed three times with sterile saline solution and air-dried. Discs were then stained with a 5% crystal violet solution (Merck, Germany) for 10 min. After staining, discs were washed with sterile saline to remove any dye excess, moved in a new microplate, and left to completely dry off. Finally, 5 ml of 99% ethanol were added to each well in order to re-solubilize the dye included in the biofilm. Crystal violet absorbance was measured at 595 nm using a microplate reader (Multiskan FC, Thermo Scientific, Italy).

### Analysis of biofilm formation by confocal laser scan microscopy (CLSM)

Sessile cultures were grown as described above. After 72 h of incubation, biofilms were gently washed with sterile saline and stained with Filmtracer™ LIVE/DEAD™ Biofilm Viability Kit (Thermo Fisher Diagnostics SpA, Rodano, Italy), according to manufacturer’s instructions. Briefly, samples were incubated with 200 μl of staining solution at room temperature in the dark for 15 min. After incubation, samples were washed with sterile saline and examined with the upright confocal laser scan microscopy TCS SP8 (Leica Microsystems CMS GmbH, Mannheim, Germany). A 488 nm laser line was used to excite SYTO9, while a 552 nm to excite propidium iodide. Sequential optical sections were collected along the *z*-axis over the complete thickness of the sample. Images from at least three randomly selected areas of each of the three discs were acquired for each disc. The obtained images were then processed with the Las X software (Leica Microsystems CMS GmbH, Mannheim, Germany) and analyzed with the Fiji software (Fiji, ImageJ, Wayne Rasband National Institutes of Health). The quantification of the fluorescence signal was performed on each independent channel following a threshold, and the mean intensity of the green and red fluorescence was measured using the Image J software, for the estimation the overall volume, along with the live/dead cells ratio [12].

### Statistical analysis

Comparisons among groups were analyzed by the Kruskal-Wallis test (GraphPad Prism v5.00 Software, USA) corrected with Dunnett’s post hoc test. All data are expressed as median and interquartile ranges. Values of *p* < 0.05 were considered as statistically significant.

## Results

### Bacterial adhesion

**Fig. 1:**
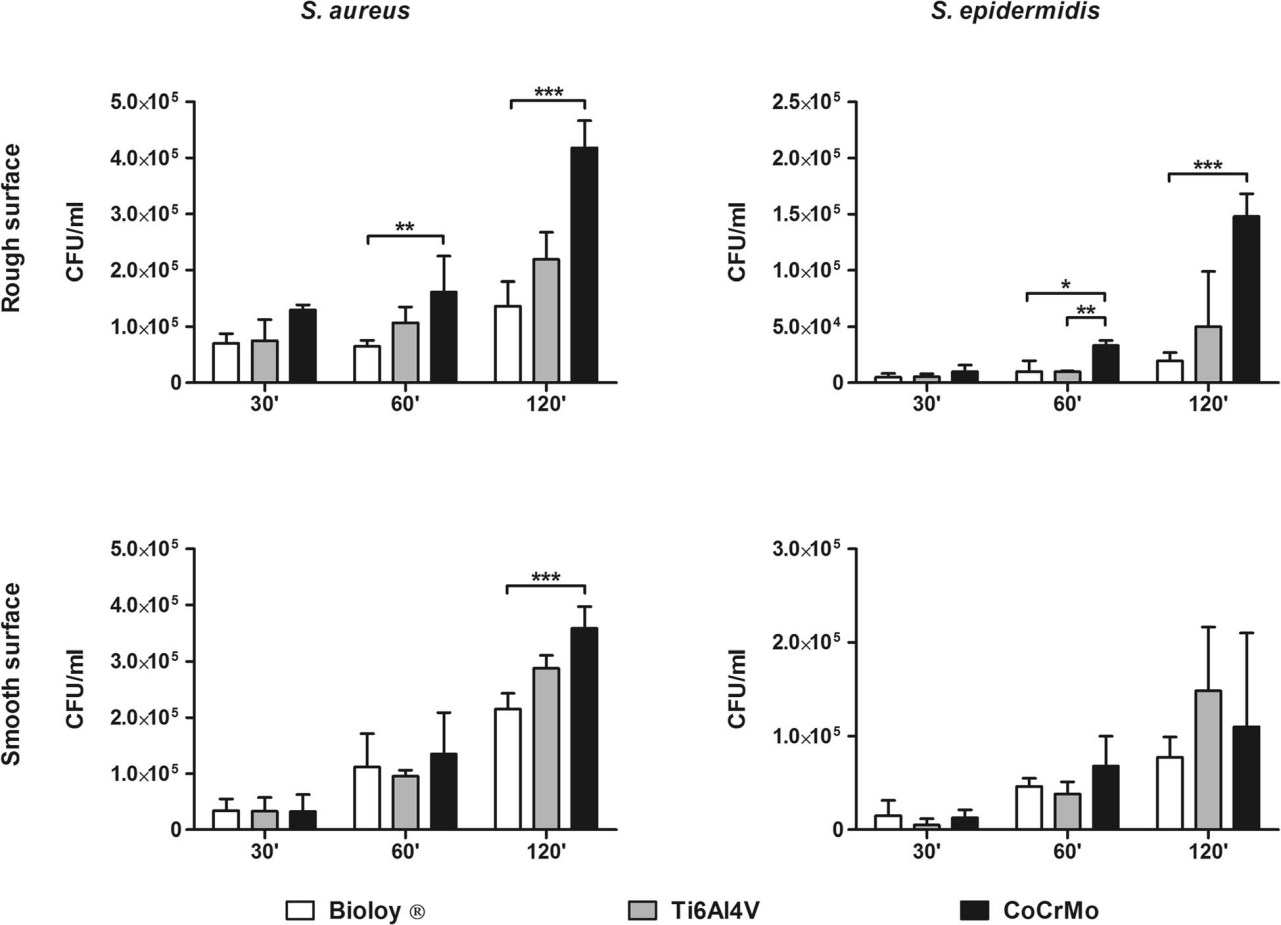
Staphylococcal cell adhesion in the first 2 h of contact with the surfaces. *Staphylococcus aureus* (left panels) and *Staphylococcus epidermidis* (right panels) adhesion on Bioloy® ceramic covered discs (Bioloy®, white bars), titanium alloy (Ti6Al4V, gray bars), and chrome-cobalt (CoCrMo, black bars). Data are expressed as median and interquartile ranges, and the statistical significance was *p < 0.05, **p < 0.01, ***p < 0.001

**Fig. 2:**
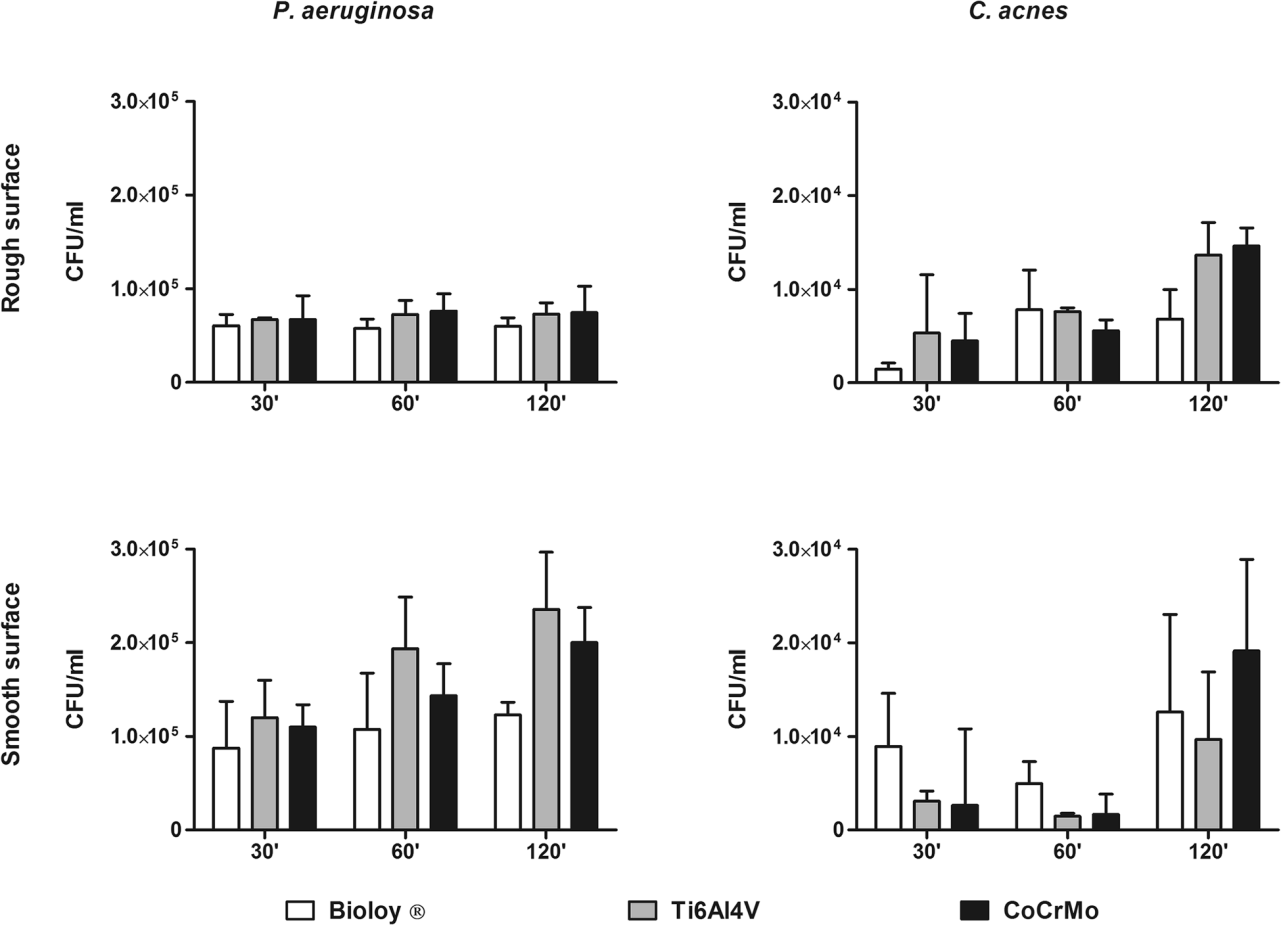
*P. aeruginosa* and *C. acnes* initial adhesion with the surfaces. *P. aeruginosa* (left panels) and *C. acnes* (right panels) adhesion on Bioloy® ceramic covered discs (Bioloy®, white bars), titanium alloy (Ti6Al4V, gray bars), and chrome-cobalt (CoCrMo, black bars). Data are expressed as median and interquartile ranges, and the statistical significance was *p < 0.05, **p < 0.01, ***p < 0.001

Bacterial adhesion on Ti6Al4V, CoCrMo, and Bioloy®-covered discs was assessed in the first hours of incubation. For what concerned staphylococci, both *S. aureus* and *S. epidermidis* displayed a statistically significant reduced adherence on Bioloy®-covered discs with respect to cobalt-chrome, with both rough and smooth surface, mainly at the later time points (Fig. 1). Adhesion to Ti6Al4V was usually comparable or slightly increases after 120 of incubation (Fig. 1). In contrast, no effects on the adhesion on either smooth or rough tested surfaces were observed for *P. aeruginosa* and *C. acnes* (Fig. 2).

### Biofilm formation

**Fig. 3:**
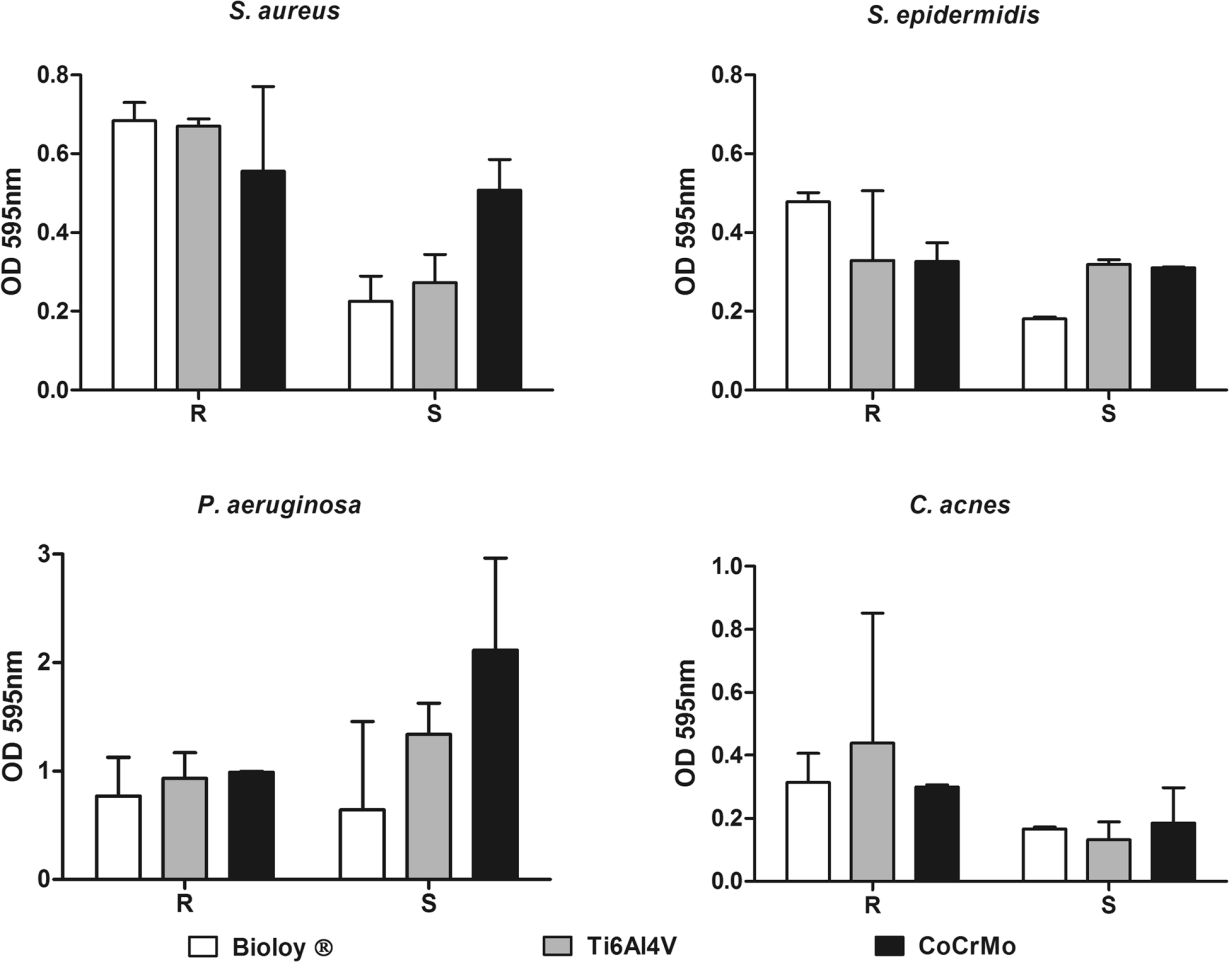
Quantification of biofilm formation by means of crystal violet assay. White bars, Bioloy®; gray bars, titanium alloy (Ti6Al4V); black bars, chrome-cobalt (CoCrMo); R, rough surface; S, smooth surface. *p < 0.05, **p < 0.01, ***p < 0.001. Data are expressed as median and interquartile ranges

Based on crystal violet staining, no significant differences in biomass could be observed for all the species (Fig. 3), although a slight reduction occurred on smooth Bioloy® discs.

**Fig. 4:**
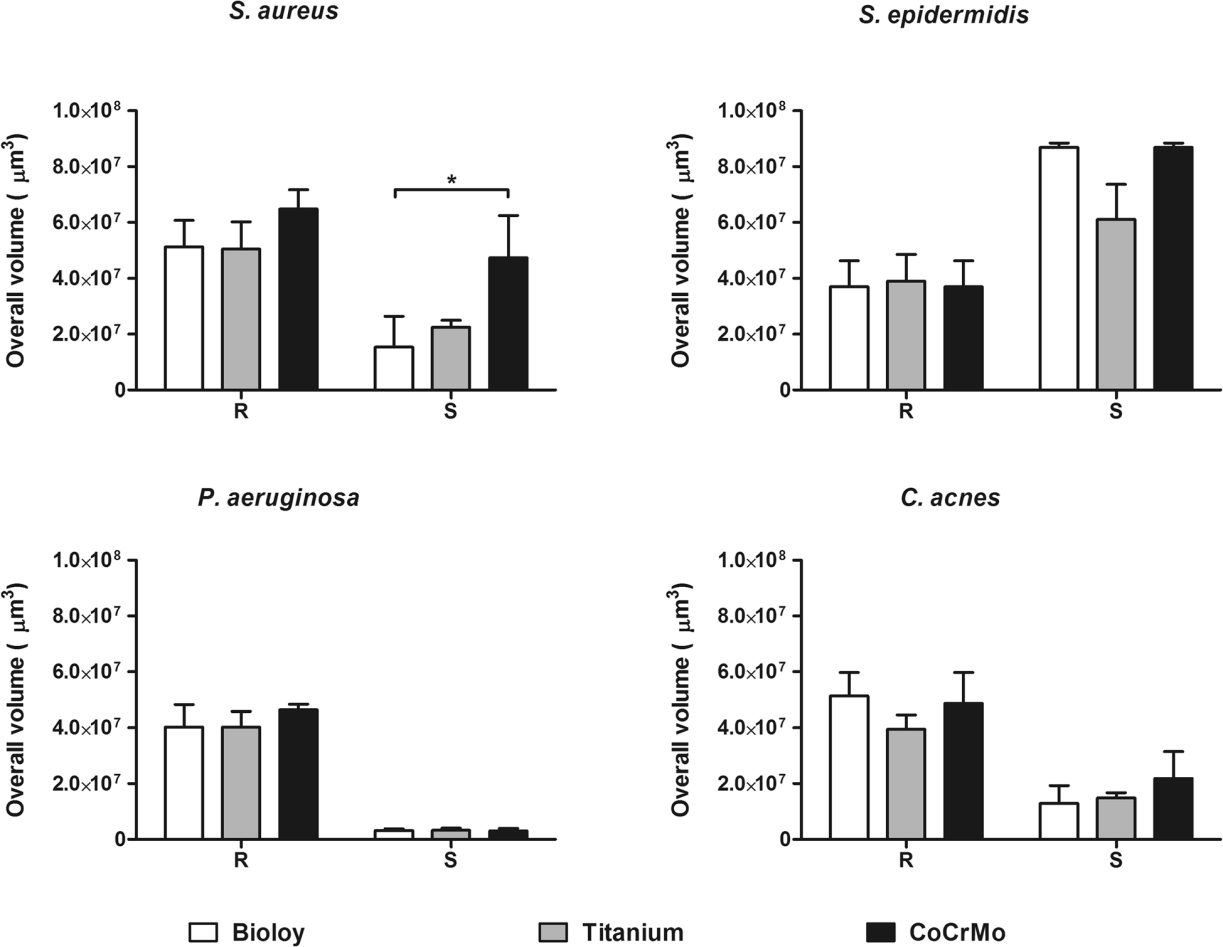
Quantification of biofilm formation by means of confocal laser scan microscopy. White bars, Bioloy®; gray bars, titanium alloy (Ti6Al4V); black bars, chrome-cobalt (CoCrMo); R, rough surface; S, smooth surface. *p < 0.05, **p < 0.01, ***p < 0.001. Data are median and interquartile ranges

**Fig. 5:**
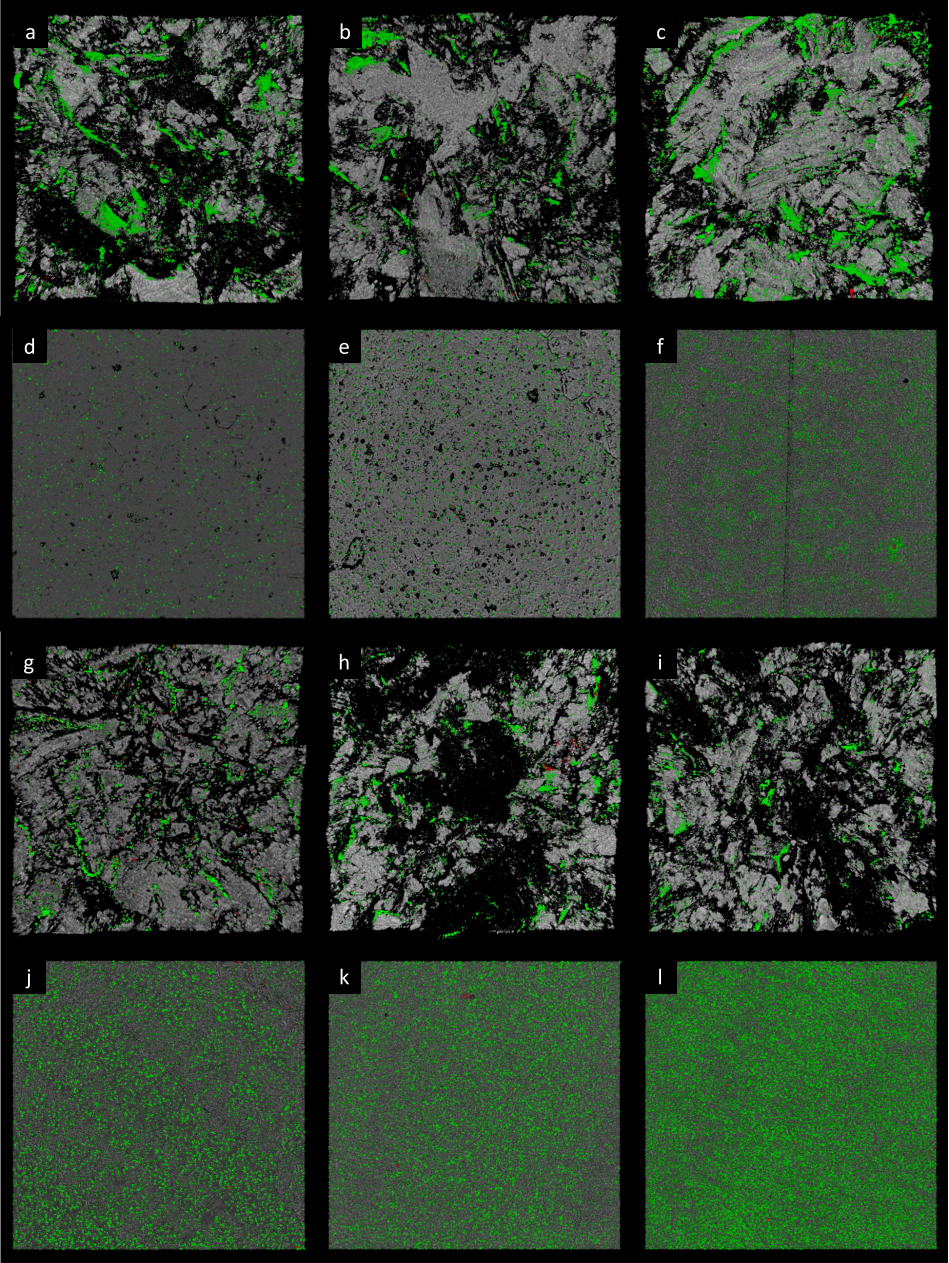
Three-dimensional reconstruction of microbial biofilm. **a**–**f***S. aureus* growth on rough **a**–**c** and smooth **d**–**f** surfaces of Bioloy® **a**, **d**, Ti6Al4V **b**, **e**, and CoCr **c**, **f**. **g**–**l***S. epidermidis* growth on rough **g**–**i** and smooth **j**–**l** surfaces of Bioloy® **g**, **j**, Ti6Al4V **h**, **k**, and CoCr **i**, **l**

**Fig. 6:**
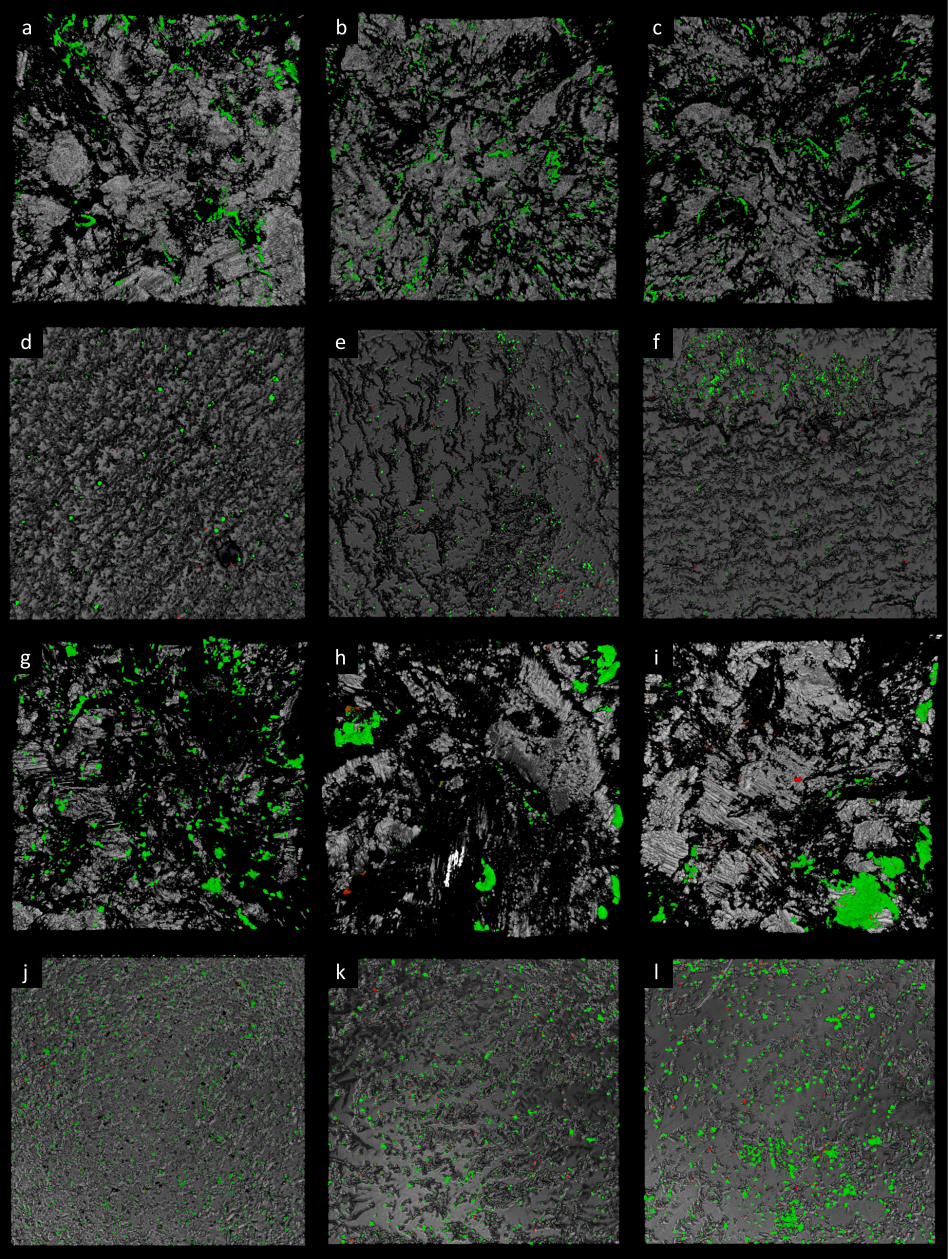
Three-dimensional reconstruction of microbial biofilm. **a**–**f***P. aeruginosa* growth on rough **a**–**c** and smooth **d**–**f** surfaces of Bioloy® **a**, **d**, Ti6Al4V **b**, **e**, and CoCr **c**, **f**. **g**–**l***C. acnes* growth on rough **g**–**i** and smooth **j**–**l** surfaces of Bioloy® **g**, **j**, Ti6Al4V **h**, **k**, and CoCr **i**, **l**

The biomass of cells embedded in biofilm was also assessed by means of CLSM. A significant reduction on TiNbN coating was only observed on mirror polished surfaces for *S. aureus* and *S. epidermidis* compared with CoCrMo (Fig. 4). No statistically significant differences were identified with regard to live/dead cell ratio (Table 1). Furthermore, macroscopic differences could not be observed in the biofilm architecture (Fig. 5 and 6), with the exception of *C. acnes*, which presented with smaller aggregates on TiNbN surfaces (Fig. 6g, j) with respect to Ti6Al4V (Fig. 6h, k) and, more markedly, to CoCrMo (Fig. 6i, l).

**Table 1:** Percentage of dead sessile cells within biofilm biomass measured by means of CLSM analysis. Significant differences are represented as follows: ^a^Bioloy® vs Ti6Al4V *p* < 0.05, ^b^CoCrMo vs Ti6Al4V *p* < 0.05

	% Dead cells in biofilm							
	*S. aureus*		*S. epidermidis*		*P. aeruginosa*		*C. acnes*	
	R	S	R	S	R	S	R	S
Bioloy®	5.9 ± 1	3 ± 1	5.2 ± 1.4	3 ± 1.8	6.7 ± 0.8	3.3 ± 0.7^a^	12.4 ± 4.1	12.3 ± 6.9
Ti6Al4V	3.5 ± 0.4	5.8 ± 1.9	6.4 ± 3.1	5.1 ± 1	3.2 ± 0.9	5.4 ± 1.5	18 ± 3.3	13.4 ± 2.9
CoCrMo	5.7 ± 2.1	3.1 ± 0.8	3.3 ± 0.9	3.1 ± 1.3	6.1 ± 3.2	3.5 ± 1^b^	13.1 ± 7.9	14 ± 5.4

*R* rough surface, *S* smooth surface

## Discussion

The introduction of a foreign body can trigger adhesion and virulence of bacteria, which is usually spontaneously cleared by host immune defenses. However, the local granulocyte function around the implant is normally impaired by the inflammatory state leading to a major susceptibility to bacterial attachment and biofilm formation, which also limit the antimicrobial therapy efficacy [6]. In this scenario, surface characteristics of the implant are crucial, and bacterial affinity towards implant surface should be carefully considered.

Ceramic coatings gained interest due to their increased hardness, surface scratch resistance, and better wettability compared with classic titanium and CoCrMo alloys, thus increasing the lubrication of the joint and decreasing the coefficient of friction and wear [13]. To manufacture this thin layer, ceramic is applied onto the whole surface of metal prosthetic components by physical vapor deposition, without implicating any modification of the underlying material properties and biomechanical functionality, only improving hardness and scratch resistance as well as providing a physical barrier against metal corrosion and metal ion’s release [14].

So far, microbiological studies on ceramic surfaces are scarce and only focused on oral streptococci adhesion on TiN-coated implants, which showed favorable effect both in in vitro and in vivo evaluations [15–19]. Indeed, a limitation of the present study is the lack of TiN-coated discs in the pool of biomaterials assayed.

In this study, we investigated the ability to adhere and form biofilm of the main etiological agents of PJIs on a commercially available TiNbN ceramic coating in comparison with Ti6Al4V and CoCrMo alloys with either rough or smooth surfaces. Overall, in the adhesion assays, aimed at simulating the first contact of bacteria with the implanted material, which encompass molecular and physical interactions, and that precede biofilm formation (cell accumulation and matrix production), the main remarkable differences were observed in *S. aureus* and *S. epidermidis.* These two species, that together account for almost 50% of all PJIs [7, 8], commonly displayed a significantly decreased attachment to Bioloy® coating after 2 h of contact, with respect to Ti6Al4V and CoCrMo. However, bacteria are very sensitive to any surface alteration (chemical or topographical), as well as to any other substance present in the physiological environment. Indeed, adsorption of proteins of the synovial joints may alter physical and chemical characteristics of such surfaces, such as wettability and free energy [15], which represent a known limitation of this in vitro study.

When the clinical isolates were tested for their ability to develop a mature biofilm on the different materials, slight differences were observed only for staphylococci grown on TiNbN, with respect to CoCrMo, while no appreciable reduction was observed with titanium alloy, which is in accordance with the only other study published so far that assessed the ability of a methicillin-resistant *S. aureus* to form biofilm on a TiNbN coating [19]. Differences in biofilm quantification could be appreciated also between the two techniques adopted in the present study. As a matter of fact, the confocal laser scan microscopy allowed only the cell biomass detection, while the crystal violet staining permitted to detect all the organic compounds, matrix comprised [20]. These could partly explain the different results obtained with the two techniques when testing *P. aeruginosa*, which owns an exceptional capability of matrix production.

Aging population is leading to a concomitant increasing demand for joint replacement procedures. Moreover, this trend is observed also in younger patients, who required prosthetic implants that should survive over the whole lifespan and withstand to higher and more frequent loads. With this dramatic growth of total joint arthroplasties, an increasing number of related complications have to be expected [21]. Besides, the most common causes of implant failure, i.e., infections, mechanical loosening, and instability [22], in the last years, the risk of developing metal hypersensitivity after joint arthroplasty is becoming a more concrete and well-defined threat [2, 23]. Even though efforts are made to improve prosthetic implant designs, to date the implant basic manufacturing raw materials are still the same of those of some decades ago, so that the underlying problem remains, and metal allergy still represents a potential and, sometimes, underdiagnosed etiology for implant failure [2].

Titanium nitride (TiN) has been the first and most known ceramic coating having a very high chemical inertness, blood tolerability, and biocompatibility with different cell type [4, 13]. In spite of being widely used for in hip and knee implants, literature reporting clinical outcomes is scarce, and the evidence of the clinical effectiveness of these ceramic coatings in terms of prevention and treatment of allergy to metal implants is still lacking [1, 4].

Titanium-niobium nitride (TiNbN) was introduced in the clinical practice to improve the physical properties of TiN, such as hardness and friction coefficient against ultra-high molecular weight polyethylene (UHMWPE) [24]. Even though conflicting results in wear testing are reported in the literature [4], TiN and TiNbN revealed a tribological behavior with UHMWPE comparable to that of CoCrMo with UHMWPE [4, 24]. TiNbN-coated implants were found to be highly non-cytotoxic and in vivo analysis on patients receiving coated dental implants showed active bone remodeling areas with new bone formation and neoangiogenesis without the presence of inflammatory infiltrate [4, 25]. Moreover, TiNbN has the ability to reduce metal ions release from the metal substrate as exhibited in vitro [26] and in vivo with hip prostheses [13].

The local metal ions released in the surrounding tissues around the metal implant could be a potential risk factor predisposing to an easier bacterial adhesion onto the prosthetic surface [27]. This concern was raised in particular for metal-on-metal total hip arthroplasty in which the amount of metal ion release can be important. A recent register study showed an increased risk of revision for periprosthetic joint infection in metal-on-metal hip arthroplasty in comparison to ceramic-on-ceramic coupling [28]. However, there is no evidence yet regarding the relation between the metal ion release and periprosthetic joint infections.

In conclusion, adhesion of some of the most common bacteria responsible for PJIs to TiNbN-coated surfaces was found to be inferior or equal to that of classical titanium and cobalt-chrome alloys. Although the onset of a PJIs is more complex than in an in vitro scenario, these findings suggest that TiNbN-coated orthopedic implants do not increase PJIs risk while ameliorating tribological and surface properties, thus representing are a valid choice to limit possible complications such as metal hypersensitivity.

## Abbreviations

BHI: Brain heart infusion broth
CLSM: Confocal laser scan microscopy
CoNS: Coagulase-negative staphylococci
PJIs: Prosthetic joint infections
SCH: Schaedler agar
SD: Standard deviation
THI: Thioglycollate broth
TiNbN: Titanium niobium nitride
TSA: Tryptic soy agar
UHMWPE: Ultra-high molecular weight polyethylene
